# Caring for Critically Ill Patients: Clinicians’ Empathy Promotes Job Satisfaction and Does Not Predict Moral Distress

**DOI:** 10.3389/fpsyg.2019.02902

**Published:** 2020-01-08

**Authors:** Giulia Lamiani, Paola Dordoni, Elena Vegni, Isabella Barajon

**Affiliations:** ^1^Department of Health Sciences, University of Milan, Milan, Italy; ^2^Prostate Cancer Program, Fondazione IRCCS Istituto Nazionale Dei Tumori, Milan, Italy; ^3^Department of Brain and Behavioural Sciences, University of Pavia, Pavia, Italy; ^4^Department of Biomedical Sciences, Humanitas University, Milan, Italy

**Keywords:** critical care, empathy, healthcare professionals, job satisfaction, moral distress, stress

## Abstract

**Background:**

Several studies have highlighted the benefits of empathy in healthcare settings. A correlation between clinicians’ empathy and patients’ adherence and satisfaction, as well as the ability for the clinician to accurately assess family members’ needs, has been found. However, empathy is often seen by clinicians as a risk factor for their wellbeing. This study aims to assess whether the level of empathy of clinicians working in critical care settings may expose them to moral distress, poor job satisfaction, and intention to quit their job.

**Methods:**

Italian clinicians who attended the 2016 “Smart Meeting Anesthesia Resuscitation in Intensive Care” completed the Empathy Quotient questionnaire, the Moral Distress Scale-Revised, and two questions assessing job satisfaction and intention to quit the job. Multiple linear and logistic regressions were performed to determine if clinicians’ empathy influences moral distress, job satisfaction, and intention to quit. Age, gender, and profession were used as control variables.

**Results:**

Out of 927 questionnaires distributed, 216 were returned (23% response rate) and 210 were used in the analyses. Respondents were 56% physicians, 24% nurses, and 20% residents. Over half of the clinicians (58%) were female. Empathy resulted the only significant predictor of job satisfaction (β = 0.193; *p* < 0.05). None of the variables included in the model predicted moral distress.

**Conclusion:**

Empathy determined neither moral distress nor intention to quit. Findings suggest that empathy is not a risk factor for critical care clinicians in developing moral distress and the intention to quit their job. On the contrary, empathy was found to enhance clinicians’ job satisfaction.

## Introduction

In psychology, the therapist’s empathy is widely acknowledged as a pivotal factor to promote patient change (Rogers, 1957). Definitions of empathy have varied (Bohart and Greenberg, 1997), though they generally have emphasized the therapist’s ability to understand the patient’s experience and feelings, and communicate this understanding to the patient (Rogers, 1957; Truax and Carkhuff, 1967). Empathy is generally described as a multidimensional construct encompassing an affective and a cognitive dimension (Baron-Cohen and Wheelwright, 2004). The affective dimension is described as feeling the patient emotions as if they were your own, but without ever losing the “as if” quality, and responding to the patient emotion with similar and appropriate emotions (Rogers, 1975). The cognitive dimension refers to the intellectual ability to understand the patient inner frame of reference, and is therefore related to the development of the theory of mind (Gladstein, 1983; Duan and Hill, 1996). Psychological literature generally distinguishes empathy from other related emotional responses, such as sympathy and personal distress. Sympathy or empathic concern has been defined as the feeling of sorrow or concern for the other person (Batson and Coke, 1983). The focus of the emotion is on the other person and differently from empathy, sympathy does not imply an exact match between one’s own emotions and the emotions of the other (Lennon and Eisenberg, 1987). Personal distress is the feeling of discomfort or self-concern that a person may feel in front of the other’s suffering. As the focus of the emotion is on the self rather than on the other, the experience of personal distress is unlikely to lead to altruistic behavior (Lennon and Eisenberg, 1987).

Several studies highlighted the benefits that empathy has for both patients and clinicians in healthcare settings. Clinicians’ empathy was found to be related to better clinical outcomes in diabetic patients (Hojat et al., 2011; Del Canale et al., 2012), increased patient satisfaction (Derksen et al., 2013), and a more accurate assessment of family members’ needs (Murphy et al., 1992; Moghaddasian et al., 2013). Other studies proved that empathy may be beneficial also for clinicians as it may promote self-efficacy and decrease burnout (Halpern, 2003; Krasner et al., 2009; Gleichgerrcht and Decety, 2013). Specifically, empathy was found to be inversely related to burnout among general practitioners (Torres et al., 2015) and medical students (Paro et al., 2014; von Harscher et al., 2017), and was found to decrease burnout and secondary traumatic stress among social workers (Wagaman et al., 2015).

Although the benefits of empathy in healthcare settings have been acknowledged, a series of studies highlighted that empathy generally diminishes during medical training as a result of the hidden curriculum and the experience in the actual healthcare environment (Hojat et al., 2009; Neumann et al., 2011). Clinicians frequently perceive empathy as a risk factor especially in those settings where closeness to death and suffering may be emotionally very difficult to manage, such as oncology, palliative, and critical care (Sanchez-Reilly et al., 2013; Riess, 2015). Clinicians may feel incapable of managing the emotions elicited in the encounter with critically ill patients (Picard et al., 2016) and therefore may prefer to engage in activities requiring little emotional investment (Hickey and Lewandowski, 1988; Stayt, 2007, 2009). This is particularly true if clinicians do not receive a training on emotions’ management or do not have the opportunity to discuss difficult cases during clinical supervisions (Berg et al., 1994).

Based on these premises, this study aims to assess whether the empathy of clinicians who work in critical care settings may be a risk factor for their psychological and occupational wellbeing. Specifically, we assessed if clinicians’ empathy predicted moral distress, poor job satisfaction, and intention to quit the job. Along with burnout, moral distress is another form of work-related distress which has been recently identified in the healthcare setting. Moral distress is the painful feeling that occurs when clinicians cannot carry out what they believe to be ethically appropriate (Lamiani et al., 2017a). Studies showed that moral distress is a relevant experience among critical care clinicians leading to depressive symptoms and job quitting (Lamiani et al., 2018). While a recent review confirmed that clinicians’ empathy is inversely correlated with burnout (Wilkinson et al., 2017), the relationship between empathy and moral distress has not been studied. Along with moral distress, we assessed the impact of clinicians’ empathy on job satisfaction and intention to quit the job. Job satisfaction and intention to quit are important outcomes of occupational wellbeing as they predict retention of healthcare professionals (Ellenbecker, 2004; De Gieter et al., 2011). Identifying factors that may promote clinicians’ job satisfaction and intention to remain is relevant for healthcare organizations which are striving to maintain a motivated workforce and reduce turnover (Lu et al., 2005).

## Materials and Methods

### Data Collection and Procedure

We conducted a cross-sectional study involving Italian critical care clinicians who attended the international conference “*Smart Meeting Anesthesia Resuscitation in Intensive Care*.” The conference was held in Milan, Italy, in May 2016. A survey composed of a series of questionnaires was developed to assess clinicians’ sociodemographic characteristics, empathy, moral distress, job satisfaction, and intention to quit the job. The survey was in Italian. Upon authorization by the Conference Scientific Committee, the survey was inserted in the Italian participants’ conference bags at registration. At the exit of the conference venue a desk was placed to collect completed surveys.

### Participants

Participants were drawn from a convenience sample of 927 Italian clinicians (614 physicians, 138 nurses, and 175 residents) who attended the conference. Of these, 216 (23%) returned the survey. Of the 216 surveys returned, 6 could not be used in the analysis. Table 1 shows the sample characteristics of the 210 surveys used in the analysis. The majority of respondents were physicians (56%) and female (58%). Participants had a mean age of 42.15 years (*SD* = 11.5) and their average working experience amounted to 11.63 years (*SD* = 9.5). Over a half of participants were married/co-habiting (69%) and lived in the north of Italy (64%). Most participants (70%) worked 36–45 h per week in mixed ICUs (70%).

**TABLE 1:** Demographic characteristics of the sample.

	**Clinicians**	
**Characteristic**	**(*N* = 210)**	**%**
**Discipline** Physician Nurse Resident Valid N	118 51 41 210	56 24 20
**Gender** Male Female Valid N	88 122 210	42 58
**Age** Mean (SD)	42.15 (11.5)	
**Years of experience** Mean (SD)	11.63 (9.5)	
**Relational state** Single Married/co-habiting Divorced Valid N	44 142 20 206	21 69 10
**Country area** North Center South Valid N	134 48 28 210	64 23 13
**Hospital beds** <300 300–600 >600 Valid N	37 73 98 208	18 35 47
**Type of ICU^∗^** Medical Post-op Neuro Mixed Other Valid N	16 17 11 144 19 207	8 8 5 70 9
**ICU Beds** ≤8 9–15 ≥16 Valid N	87 97 22 206	42 47 11
**Working hours per week** ≤35 36–45 ≥45 Valid N	11 146 52 209	5 70 25

*^∗^ICU, intensive care unit.*

### Ethics Statement

As the study did not involve patients and was purely observational, ethical approval was not required as per applicable institutional and national guidelines and regulations. The Scientific Committee of the SMART conference approved the survey administration. The study has been carried out in accordance with the Code of Ethics of the Declaration of Helsinki. All participants provided written informed consent granting permission to use the data for research purposes. The surveys were completely anonymous.

### Measures

#### Empathy

The Italian validated version (Preti et al., 2011) of the empathic quotient (EQ) questionnaire was used to measure empathy. The EQ is a self-report questionnaire originally developed by Baron-Cohen and Wheelwright (2004) to provide a global measure of empathy, comprising the affective and cognitive dimensions (Lawrence et al., 2004). According to the authors of the measure, empathy is a multidimensional construct combining the ability to feel an appropriate emotion in response to another’s emotion and the ability to understand the others’ emotion (perspective-taking) and behave accordingly. Consistent with the theoretical framework of empathy, factor analysis revealed that the construct of empathy measured by the EQ comprises three factors: cognitive empathy, emotional reactivity, and social skills (Preti et al., 2011). However, a second-order factor analysis confirmed that the EQ scale has been developed to provide a global measure of empathy and many studies using EQ commonly report global scores (Baron-Cohen and Wheelwright, 2004; Bangash et al., 2013; Lachmann et al., 2018). The EQ is composed of 40 items measuring empathy and 20 filler-items. Responses to items are given on a four-point Likert scale ranging from 1 (strongly agree) to 4 (strongly disagree). Participants receive 0 for a non-empathic response, whatever the magnitude, and 1 or 2 for an empathic response depending on the strength of the reply. Responses on filler items are not included in the score counting. The EQ total score ranges from 0 to 80 with higher scores indicating a higher level of empathy. As we were interested in the global impact of empathy on clinicians’ wellbeing, the total empathy score was used in the analysis.

The Italian EQ has good internal consistency (Cronbach’s α = 0.79) and reliability (test–retest at 1 month Pearson’s *r* = 0.85) (Preti et al., 2011). In this study, Cronbach’s α-value for the EQ was 0.85.

#### Moral Distress

The Italian validated version of the Moral Distress Scale-Revised (MDS-R) (Hamric et al., 2012) was used to measure moral distress. The Italian MDS-R presented good reliability and psychometric properties (Lamiani et al., 2017b). The Italian MDS-R is composed of 14 items describing morally distressing situations. For each item, participants have to fill out a frequency scale, which assesses how often the situation is experienced, and an intensity scale, which measures how disturbing the situation is. Responses are given on a five-point Likert scale ranging from 0 (never) to 4 (very frequently) for the frequency scale, and from 0 (none) to 4 (great extent) for the intensity scale. The total MDS-R is obtained by summing the frequency × intensity scores and dividing the total by the number of items. The total score ranges from 0 to 16 with higher scores indicating a greater degree of moral distress. The Italian MDS-R scale has good internal consistency (Cronbach α = 0.81). In this study, Cronbach’s α-value for the MDS-R was 0.81.

#### Job Satisfaction

Job satisfaction was measured through the widely used one-item scale from Aiken et al. (2002) ranging from 1 (very unsatisfied) to 4 (very satisfied). This item has been used to assess job satisfaction in previous studies (Aiken et al., 2002; Dordoni et al., 2019).

#### Intention to Quit

Intention to quit was measured through the one-item question which is generally included at the end of the MDS-R. The question inquires if the person has ever thought of leaving or change his/her working position. The questions had two options (1 = No, I have never thought of leaving my position; 2 = Yes, I thought of leaving my position or I have already quit). This item has been used in several studies on moral distress (Hamric and Blackhall, 2007; Hamric et al., 2012).

### Statistical Analysis

Statistical analysis was conducted using SPSS software for Window (22 version). First, descriptive analyses were conducted. Second, correlation analysis was run in order to examine bivariate correlations between study’s constructs and control variables (gender, age, discipline, working hours per week, and years of working experience). Multiple linear regression analyses were then conducted to investigate whether EQ predicted job satisfaction and moral distress. Multiple logistic regression was conducted to verify if the EQ predicted the intention to quit. In the regression models, we entered some covariates that were found in the literature to be significantly correlated with the study variables (gender, age, discipline, working hours per week, and years of working experience).

## Results

### Relationships Between Empathy, Moral Distress, Job Satisfaction, and Intention to Quit

Table 2 shows means, standard deviations, and correlations between the study variables. Slightly over half of the participants (62%) stated that they would not quit their job, whereas 38% referred that they had thought of or had actually quit their job. Significant correlations were found between intention to quit and job satisfaction (*r* = −0.18, *p* < 0.01), and between intention to quit and moral distress (*r* = 0.33, *p* < 0.01). EQ was found to be related to job satisfaction (*r* = 0.17, *p* < 0.05).

**TABLE 2:** Mean, standard deviation, and correlation between study variables (*N* = 210).

**Variable**	**Mean (SD)**	**1**	**2**	**3**	**4**	**5**	**6**	**7**	**8**	**9**
1. Empathic quotient	42.58 (10.84)	–	–0.28	0.17^∗^	–0.08	0.42^∗∗^	–0.09	–0.095	–0.004	–0.073
2. Moral distress	5.04 (2.36)		–	–0.10	0.33^∗∗^	0.13	–0.11	–0.148	0.108	0.034
3. Job satisfaction	2.81 (0.73)			–	–0.18^∗∗^	0.02	−0.14^∗^	–0.115	0.072	0.018
4. Intention to quit	–				–	0.05	0.17^∗^	0.131	–0.087	0.004
5. Gender (female)	–					–	–0.08	–0.042	0.170^∗^	–0.121
6. Age	42.15 (11.5)						–	0.877^∗∗∗^	–0.654^∗∗∗^	0.025
7. Years of experience	11.63 (9.5)							–	–0.510^∗∗∗^	–0.032
8. Discipline (physician)	–								–	–0.019
9. Working hours per week	–									–

*^∗^*p* < 0.05; ^∗∗^*p* < 0.01; ^∗∗∗^*p* < 0.001.*

Stepwise multiple linear regression analysis was performed to explore the predictors of job satisfaction and moral distress (Table 3). EQ resulted to be the only significant predictor of job satisfaction (β = 0.193; *p* < 0.05), even when checking for covariates. None of the variables included in the model predicted moral distress.

**TABLE 3:** Stepwise multiple regression predicting job satisfaction and moral distress (*N* = 210).

	**Job satisfaction**			**Moral distress**		
	***B***	**β**	***p*-value**	***B***	**β**	***p*-value**
**Model 1**						
Age	–0.012	–0.182	0.305	–0.010	–0.047	0.803
Gender (female)	–0.002	–0.001	0.987	0.510	0.107	0.207
Discipline (physician)	–0.056	–0.060	0.564	–0.023	–0.008	0.942
Years of experience	0.002	0.031	0.846	–0.043	–0.174	0.305
Working hours per week	0.107	0.075	0.344	–0.046	–0.010	0.903

*R*^2^	0.021			0.032		

**Model 2**						
Age	–0.012	–0.181	0.302	–0.009	–0.043	0.817
Gender (female)	–0.131	–0.087	0.320	0.757	0.159	0.091
Discipline (physician)	–0.23	–0.025	0.812	–0.022	–0.007	0.946
Years of experience	0.005	0.068	0.668	–0.047	–0.190	0.263
Working hours per week	0.106	0.074	0.342	–0.025	–0.005	0.948
Empathic quotient	0.013	0.193	0.025	–0.024	–0.117	0.203

*R*^2^	0.051			0.043		

Multiple logistic regression models were run in order to test the effect of EQ on intention to quit the job (Table 4). Results showed no effect of EQ on intention to quit.

**TABLE 4:** Multiple logistic regression of empathic quotient on intention to quit (*N* = 210).

	**β (SE β)**	**Wald**	***p*-value**	**OR**	**95% CI**
Age	0.008 (0.030)	0.074	0.786	1.025	0.962–1.092
Gender (female)	0.249 (0.312)	0.639	0.424	1.508	0.721–3.155
Discipline (physician)	0.020 (0.269)	0.006	0.940	1.050	0.613–1.801
Years of experience	0.020 (0.033)	0.365	0.546	1.001	0.936–1.072
Working hours per week	0.041 (0.293)	0.019	0.889	0.959	0.517–1.781
Empathic quotient	−0.019 (0.017)	1.210	0.419	0.981	0.949–1.015

## Discussion

Clinicians working in critical care settings are often exposed to emotionally and ethically challenging clinical situations (Donchin and Seagull, 2002). Recent studies showed that working in critical care settings may expose clinicians to stress-related conditions such as burnout, secondary traumatic stress, moral distress, and depression (Embriaco et al., 2007a, b, 2012; Berg et al., 2016; Moss et al., 2016; Lamiani et al., 2018). A common belief among clinicians is that being empathic could make them more vulnerable to the patients’ and families’ suffering and therefore may be a risk factor for their emotional wellbeing (Tanriverdi, 2013; Kerasidou and Horn, 2016). This study is the first to assess if empathy of clinicians working in a critical care setting could negatively affect their psychological and occupational wellbeing. Specifically, we assessed if empathy predicted moral distress, poor job satisfaction, and intention to quit the job.

Our findings suggest that empathy is not a risk factor for critical care clinicians’ wellbeing, as it does not predict moral distress or the intention to quit their job. On the contrary, empathy was found to enhance job satisfaction.

Specifically, we found that empathy does not predict moral distress nor correlates with it. Our findings suggest that empathy, as the capacity to perceive the internal frame of reference of another person with its emotional components (Rogers, 1959), may not be linked or lead to an increased violation of the clinician’s moral integrity. In other words, understanding and emotionally responding to the point of view of the other – be it a patient or a colleague – does not imply condescending to undertake professional actions that are perceived by clinicians as morally inappropriate. Consistently, the literature has increasingly acknowledged the need for clinicians to cultivate both empathy and moral resilience as pivotal qualities in patient care (Kerasidou and Horn, 2016; Rushton, 2016). Empathy may be accompanied by assertiveness and responsibility regarding the moral choices to carry on professionally, even if these may entail disagreements and misalignments with family members or colleagues (Halpern, 2007; Roeland et al., 2014). In the recent literature, this quality has been defined as “moral resilience.” Moral resilience is the capacity of an individual to sustain or restore his/her integrity in response to moral complexity, confusion, distress, or setbacks (Rushton, 2016). Moral resilience involves choosing how to respond to ethical challenges and uncertainty in ways that preserve one’s own integrity, minimize suffering, and allow to serve patients with highest purpose (Rushton, 2016).

Caring for critically or terminally ill patients often brings up the issue of clinicians’ emotional involvement and the related fear of being overwhelmed by the patients’ suffering. The common belief that empathy may be a risk factor for clinicians’ wellbeing probably lays in the confusion between empathy and sympathy (Kerasidou and Horn, 2016; Thirioux et al., 2016). Unlike sympathy, which entails an identification between self and the other (Thirioux et al., 2016), empathy implies the ability to enter the world of the other without losing the boundaries of the self and without confounding or identifying with the patient (Rogers, 1980). Empathy, therefore, is the ability to understand and feel the perspective of the other, without projecting one’s own emotions onto the other (Kerasidou and Horn, 2016). Despite some studies highlighted the positive effects of sympathy in promoting altruistic behaviors (Batson et al., 1987), other studies in the healthcare field showed that sympathy, rather than empathy, could expose clinicians to secondary traumatic stress (Crumpei and Dafinoiu, 2012) and lead to a disproportionate use of clinical resources (Nightingale et al., 1991). Crumpei and Dafinoiu (2012) found that sympathetic clinicians were more vulnerable to secondary traumatic stress than empathic clinicians, who did not report traumatic symptoms. Nightingale et al. (1991) found that sympathetic physicians, on average, had a greater preference for intubation, ordered more laboratory tests, and performed cardiopulmonary resuscitation for longer periods of time before declaring their efforts unsuccessful compared to empathic physicians. The literature shows that also the opposite tendency of sympathy, which is called alexithymia, may be problematic for clinicians’ wellbeing. Alexitimia is the difficulty to identify one’s own and the other’s emotions, which results in the tendency to ignore those emotions (Thirioux et al., 2016). Alexitimic traits were found to be associated with burnout and secondary traumatization among physicians (Gleichgerrcht and Decety, 2013). Specifically, Gleichgerrcht and Decety (2013) found that physicians who had difficulty in identifying emotions and regulating their negative arousal developed emotional exhaustion, detachment, and a low sense of accomplishment. Empathy, as the middle way between emotional over- and under involvement, seems protect clinicians from burnout and secondary traumatic stress (Torres et al., 2015; Wagaman et al., 2015). Consistently with the literature, our findings provide evidence that empathy is not a risk factor for developing moral distress.

Based on this study’s findings, we may hypothesize that other psychological factors, rather than empathy, may contribute to the development of moral distress, such as sympathy, lack of assertiveness, poor self-esteem, or lack of flexibility. As it happens in many stress-related conditions, also organizational factors may play an important role in contributing to moral distress, such as poor ethical climates, management styles, and working atmosphere. Further research should be conducted to assess these hypotheses.

In this study, we found that empathy does not predict clinicians’ intention to quit their job. On the contrary, it increases job satisfaction. In other words, more empathic clinicians reported being more satisfied with their job. Probably, the ability to feel and understand the perspective of the other allows clinicians to tailor their behaviors, negotiate different expectations, and therefore build more positive and satisfactory relationships with patients and colleagues. In the helping professions, where relationships play an important role, being empathic and thus being able to connect with patients without being overwhelmed, could constitute a source of job satisfaction. In the face of medicine’s limits and treatment failures, providing empathy to patients is sometimes the only source of meaning and healing (Sinclair et al., 2017).

This study has several limitations related to the research design and methodology. For what concerns the research design, as the literature lacks a model on moral distress, no underlying theory guided the choice of moral distress predictors. We explored the role of empathy, but we did not assess the role of other individual and organizational variables such as sympathy, assertiveness, and ethical climates in determining moral distress. Moreover, the use of the total empathy scores instead of the subscales’ scores may have covered the effect of emotional reactivity and cognitive empathy on moral distress, job satisfaction, and intention to quit. Due to the small sample size, no moderation nor mediation analyses have been conducted although these could have been informative. Even if in this study a direct relationship between empathy and moral distress was not found, future studies could assess the moderator or the mediator role of assertiveness and ethical climates. In addition, we assessed the effects of empathy only on moral distress, job satisfaction, and job quit, which are limited indicators of clinicians’ psychological and occupational wellbeing. For what concerns the methodology, our participants were drawn from a convenience sample of Italian clinicians who voluntarily completed the survey. This, along with the low response rate, may have introduced a self-selection bias and therefore the generalizability of our findings is limited. The findings are based on self-reported measures and therefore are subject to self-reported measures biases. Finally, the data were cross-sectional. Therefore, our conclusions have to be interpreted with caution, especially for what concerns the direction of causality.

Despite these limitations, our findings have important practical implications for healthcare organizations and for clinicians working in intensive care units. Empathy, as a protective factor for clinicians’ psychological and occupational wellbeing, should be cultivated and incorporated in the training of critical care clinicians. As empathy develops through experience and by increasing self-awareness of one’s identity and personal values and boundaries (Davis, 1990), it cannot be directly taught. Its development, however, can be cultivated and promoted by providing clinicians with experiential opportunities and appropriate resources. Clinical supervisions facilitated group discussions, simulated training programs with actors’ feedback and mindfulness training programs (Epstein et al., 2008; Sorensen and Iedema, 2009; Bell et al., 2014) can help clinicians to develop empathy by promoting self-reflection and opening up to different perspectives on patients care.

## Data Availability Statement

The data that support the findings of this study are not publicly available. The informed consent signed by participants did not include the possibility to distribute the data to third parties.

## Ethics Statement

As the study did not involve patients and was purely observational, ethical approval was not required as per applicable institutional and national guidelines and regulations. The Scientific Committee of the SMART conference approved the survey administration. The study has been carried out in accordance with The Code of Ethics of the Declaration of Helsinki. All participants provided written informed consent granting permission to use the data for research purposes. The surveys were completely anonymous.

## Author Contributions

GL conceived and designed the research, supervised the work, acquired the data, analyzed and interpreted the data, and drafted and critically revised the manuscript. PD analyzed and interpreted the data, and drafted and critically revised the manuscript. EV conceived the research project, interpreted the data, and critically revised the manuscript. IB interpreted the data and critically revised the manuscript.

## Conflict of Interest

The authors declare that the research was conducted in the absence of any commercial or financial relationships that could be construed as a potential conflict of interest.
